# One-stage urethroplasty using a combination of buccal mucosa graft and Q penile skin flap for a complicated urethral stricture: A challenging case report

**DOI:** 10.1097/MD.0000000000041888

**Published:** 2025-03-21

**Authors:** Abdulrazak Anadani, Aya Obaidin, Bashar Badawi, M. Yasin Lutfi

**Affiliations:** a Faculty of Medicine, University of Aleppo, Aleppo, Syria; b Faculty of Medicine, Hama University, Hama, Syria; c Clinical Specialist in Urology, Hama National Hospital, Hama, Syria.

**Keywords:** buccal mucosa graft, case report, penile skin flap, Q flap, surgical complications, urethroplasty

## Abstract

**Rationale:**

Strictures of the male urethra are common, often caused by trauma or occur idiopathically. The primary clinical symptoms include chronic obstructive voiding issues, and some patients may experience sexual dysfunction. Treatment choices mainly depend on the stricture’s location and length.

**Patient concerns:**

A 37-year-old man visited the urology department with lower urinary tract symptoms and recurrent urinary tract infections. He had a 5 cm urethral stricture previously treated with urethroplasty, which was unsuccessful.

**Diagnoses:**

A retrograde urethrography revealed the stricture, an extra-anatomical bypass, and a diverticulum.

**Interventions:**

A second urethroplasty was performed, using a combination of a buccal mucosa graft and a penile skin flap.

**Outcomes:**

Postoperative follow-up indicated improvement in the patient’s voiding symptoms. A retrograde urethrography was done 3 months after the procedure showing a well-patent urethra with no complications.

**Lessons:**

The key factors in deciding the treatment for urethral strictures are their location and length. Both grafts and flaps are effective for urethroplasties. However, complex long strictures with damaged urethral plates pose challenges for successful single-stage reconstruction. Combining a dorsal buccal mucosa graft to augment the urethral plate with a ventral onlay penile skin flap is a promising approach, leveraging the benefits of both tissue types. Combining grafts and flaps is advisable for reconstructing complicated urethral strictures with damaged urethral plates. Consulting a more experienced surgeon is recommended to minimize the risk of complications.

## 1. Introduction

Strictures of the male urethra are relatively common. The most frequent etiologies are trauma and idiopathic. Traumatic strictures can be iatrogenic, such as oversized rigid endoscopy, previously performed endoscopic procedures on the bladder or the prostate and traumatic placement of urinary catheters, or sexually-induced, such as penile fractures. Other etiologies may be sexually transmitted infections, radiation therapy, and inflammatory skin conditions such as lichen sclerosus. The main etiology remains unknown in many cases because of the lag time between exposure and the manifestation of stricture.^[1,2]^

The most likely clinical manifestations are chronic obstructive voiding symptoms such as decreased urinary stream, incomplete emptying of the bladder, and in some cases acute urinary obstruction. Recurrent urinary tract infections can be caused by the urinary stasis and therefore dysuria, urinary spraying and other lower urinary tract symptoms can exist. Some patients may have sexual dysfunction.^[3]^

Cystourethroscopy, retrograde urethrogram (RUG), voiding cystourethrogram, and ultrasound urethrography are all useful investigations to confirm the diagnosis.^[2]^

Treatment options include minimally invasive therapies such as dilatation and direct vision internal urethrotomy (DVIU), and surgical urethroplasty using autologous grafts, skin flaps or a combination of both. The choice of treatment approach depends mainly on the location and the length of the stricture.^[2]^

In this paper, we report a successful single-staged urethroplasty with the combination of both buccal mucosa graft and Q penile skin flap in a 37-year-old male with a 5 cm long anterior urethral stricture, diverticulum and extra-anatomical bypass after an unsuccessful previous urethroplasty.

## 2. Case presentation

**Figure 1.**
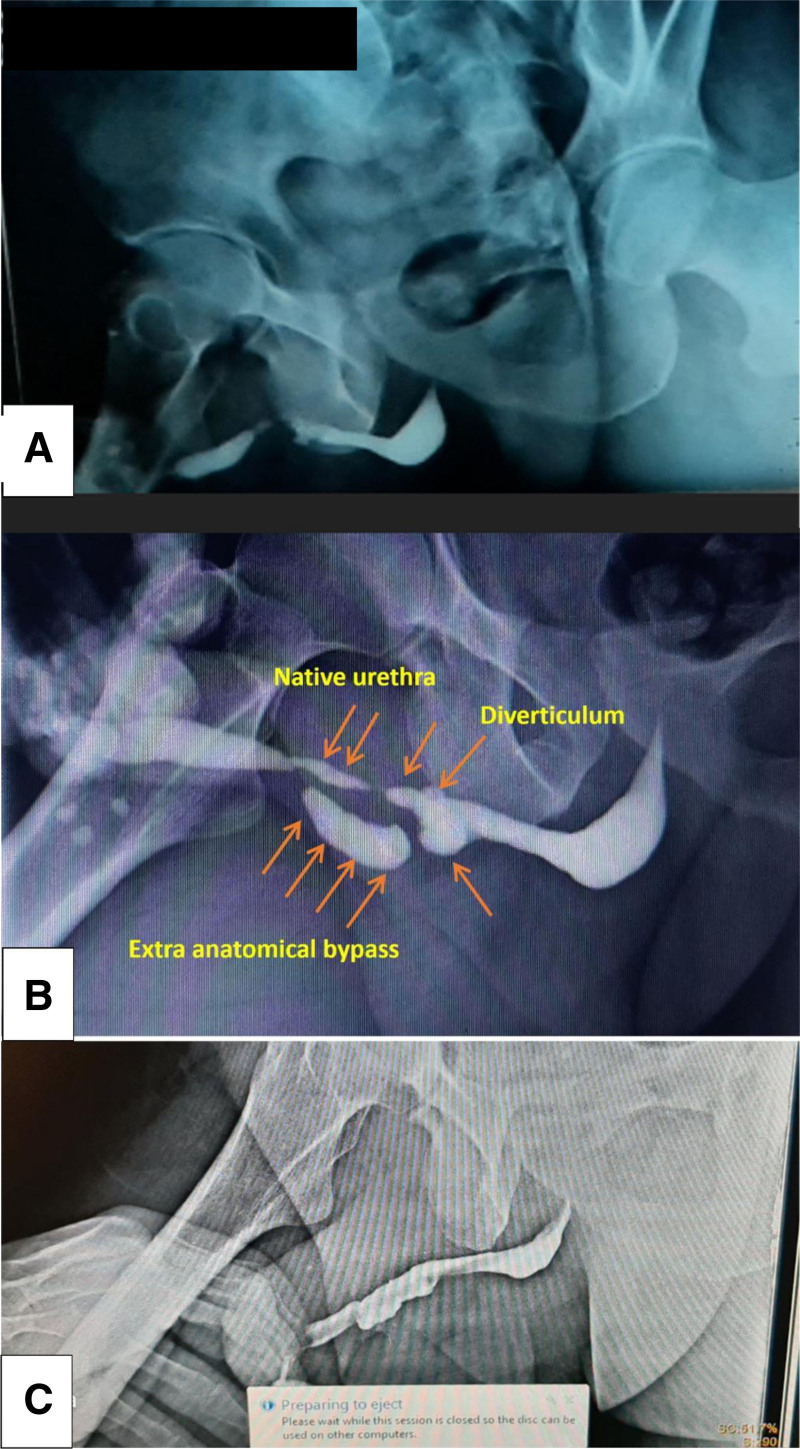
(A) A RUG at patient’s first presentation in 2019 shows a 5 cm anterior urethral stricture. (B) A RUG after the unsuccessful urethroplasty shows the urethral stricture, an extra-anatomical bypass and a diverticulum. (C) A RUG 3 months after the second urethroplasty. RUG = retrograde urethrogram.

**Figure 2.**
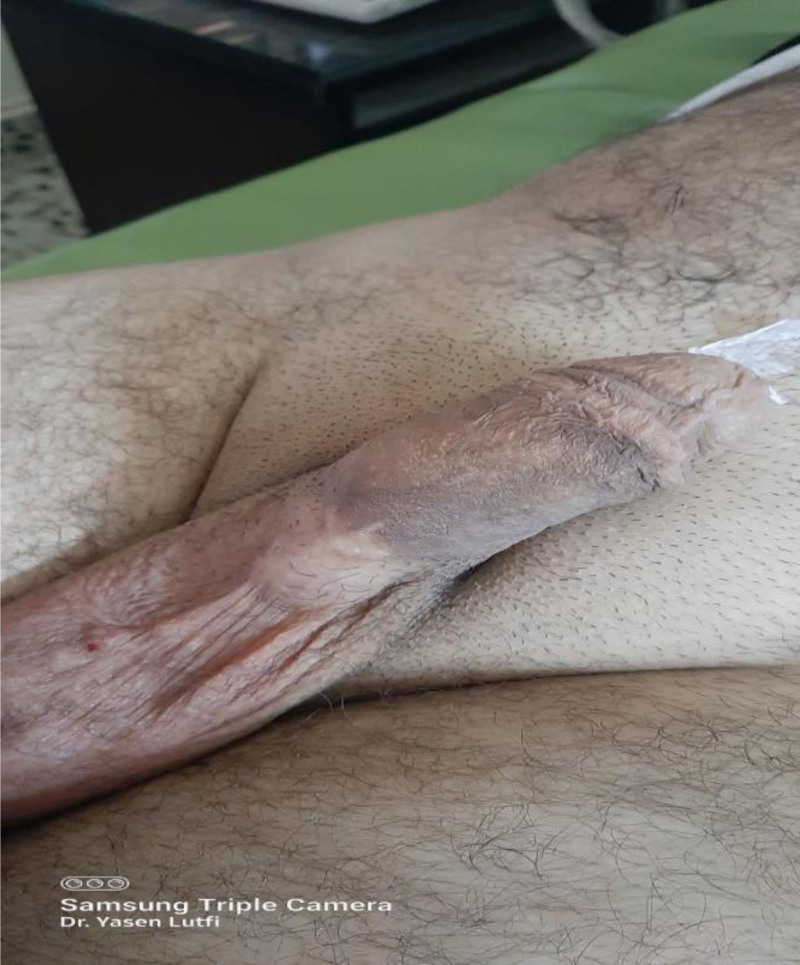
The penoscrotal region shows a bizarre mobile superficial channel.

A 37-year-old man presented to the Department of Urologic Surgery with lower urinary tract symptoms and recurrent urinary tract infections. In clinical history, the patient was diagnosed with urethral stricture in 2019 (Fig. 1A). He underwent 2 attempts of DVIUs which were not beneficial. In 2021, the decision of urethroplasty was made, which was unsuccessful due to its inappropriate technique; surgeons used a scrotal flap to create an extra-anatomical bypass without overlapping the urethral plate. There was no other remarkable medical or drug history. Clinical examination of the penoscrotal region showed a bizarre mobile superficial channel created in the previous urethroplasty (Fig. 2).

A RUGshowed a 5 cm long pendulous urethral stricture, an extra-anatomical bypass and a diverticulum (Fig. 1B).

**Figure 3.**
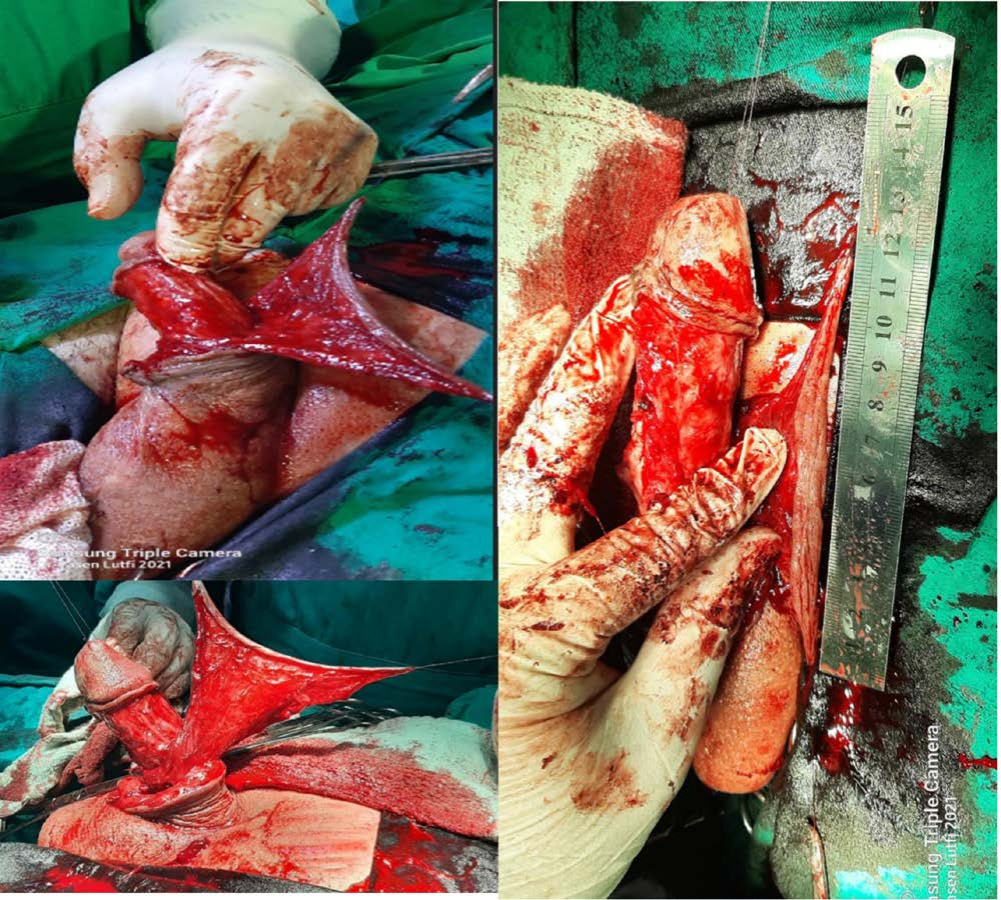
Harvesting a circumferential penile skin flap with dorsal pedicled extension (Q flap).

**Figure 4.**
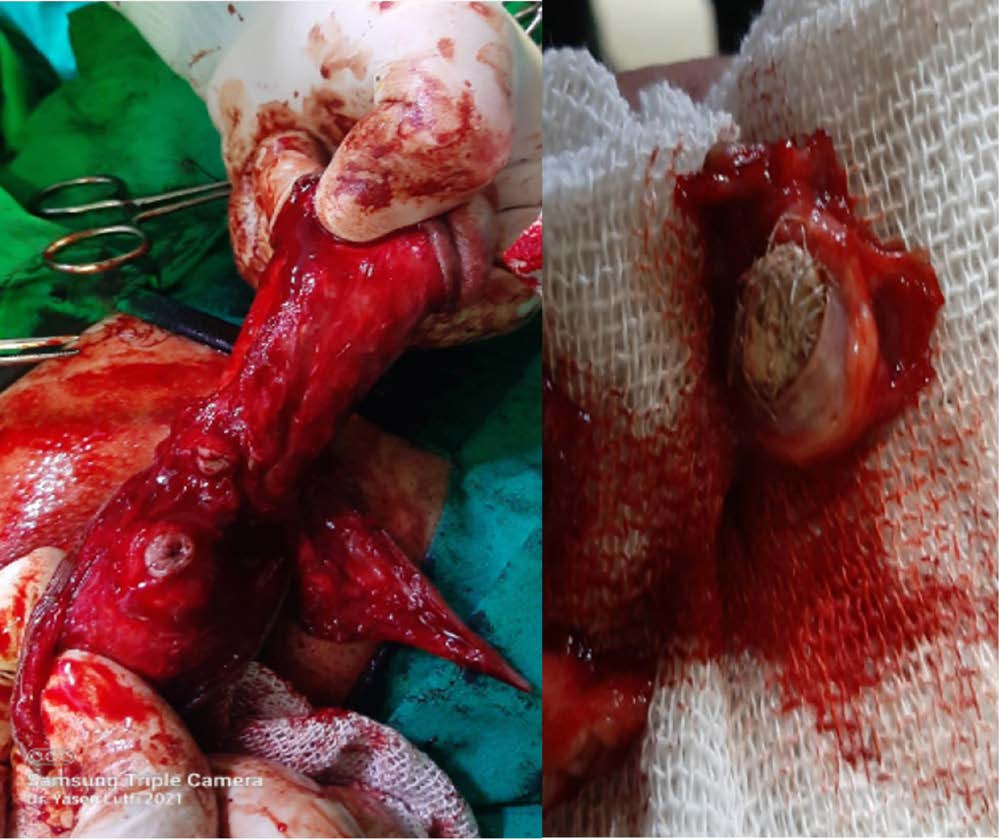
Excision of the extra-anatomical hairy penoscrotal bypass and the diverticulum which was also filled with hair.

The decision to do a 1 stage urethroplasty was made. After general anesthesia and sterilization, distal circumferential penile skin incisions were made to harvest a circumferential skin flap with dorsal pedicled extension (Q flap; Fig. 3). Then the extra-anatomical hairy penoscrotal bypass and the diverticulum were resected (Fig. 4).

**Figure 5.**
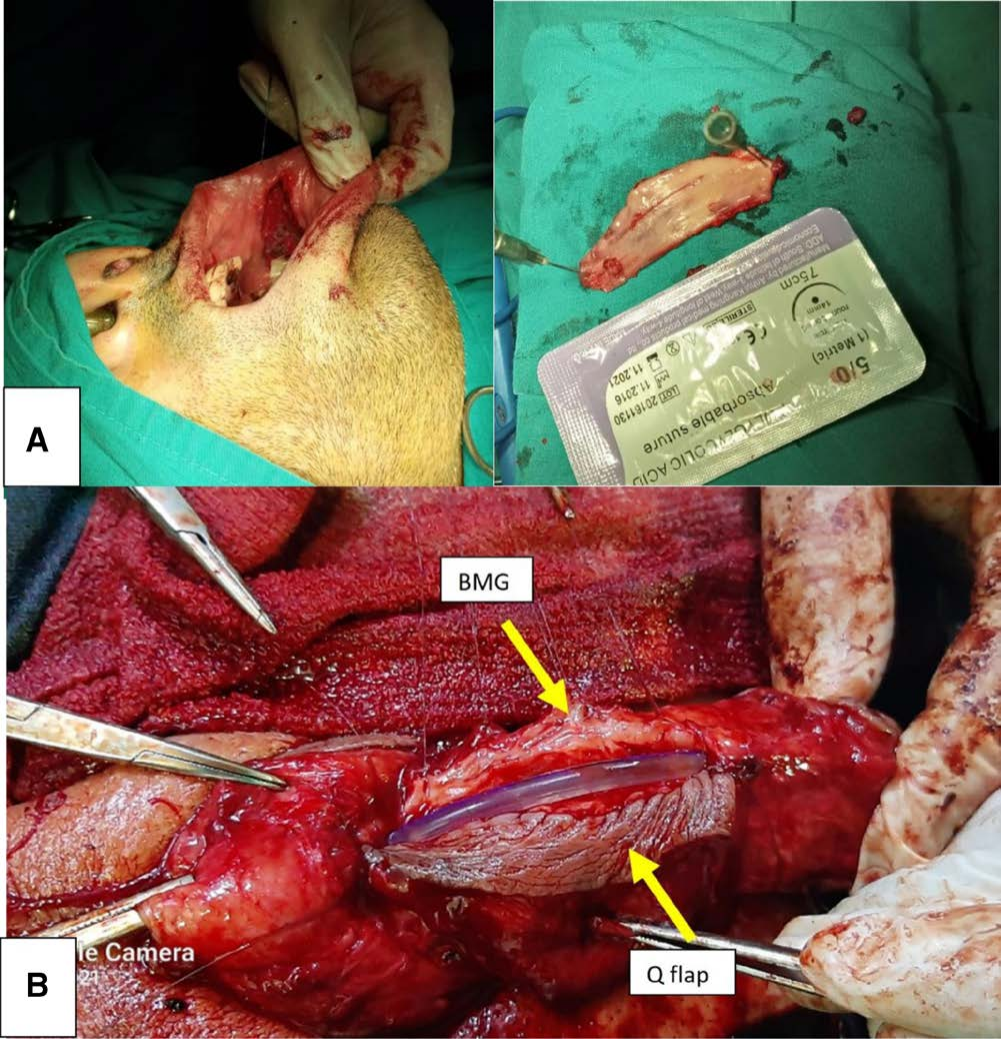
(A) Harvesting a non-closure buccal mucosa graft (BMG) with nasal intubation. (B) The BMG was dorsally attached to the damaged urethral plate and the Q flap was ventrally sutured. BMG = buccal mucosa graft.

**Figure 6.**
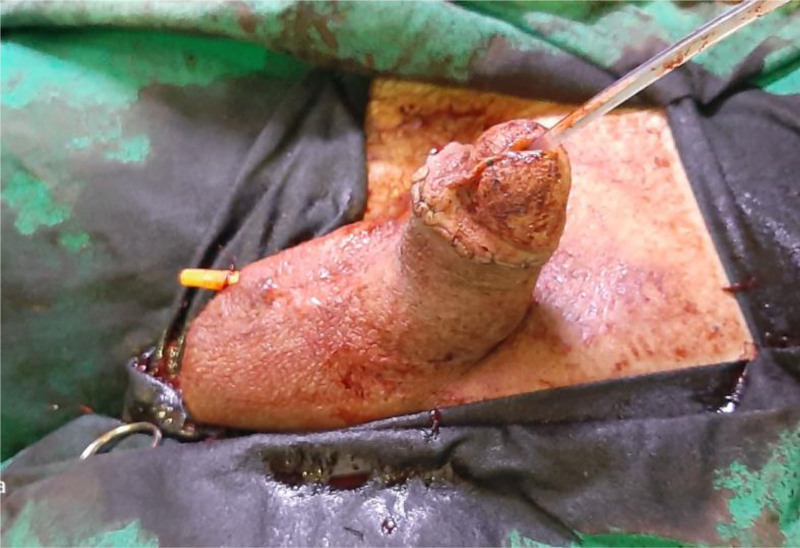
The distal circumferential penile incision was closed and a 14 Fr urinary catheter with a drain was inserted.

Ventral dissection of the urethra exposed 7 cm of damaged urethral plate which indicated harvesting a buccal mucosa graft (BMG) to enhance the urethral plate (Fig. 5A). After confirming the urethral patency in both proximal and distal directions, the BMG was dorsally attached to the urethral plate using 4/0 vicryl to suture the margins, and followed by the onlay ventral suturing of the Q flap (Fig. 5B). Finally, the distal circumferential penile incision was closed and a 14 Fr urinary catheter with a drain was inserted (Fig. 6).

Prophylactic antibiotics were administered and the patient was discharged the next day. The patient was assessed weekly and reviewed for dressing and the catheter was removed on day 30. The patient’s voiding symptoms improved and a RUG was done 3 months after the procedure showing a well-patent urethra. no diverticula or other anatomical abnormalities were observed. And there was no evidence of contrast extravasation (Fig. 1C).

## 3. Discussion

Over the years, more surgical techniques regarding urethroplasty are being developed, and keeping up with this information is a must for the urologist.^[1]^ In order to ease the description of the male urethra, it has been divided into 2 main parts: the anterior urethra which includes the penile and the bulbar urethra, and the posterior urethra which includes the prostatic and membranous urethra.^[4]^

The management of urethral strictures includes minimally invasive techniques such as dilation, DVIU, or urethroplasty.

Dilation and DVIU are recommended under the condition of having a <2 cm stricture in the bulbar urethra and in meatal or fossa navicularis strictures. On the other hand, urethroplasty is recommended for penile strictures, >2 cm bulbar strictures and recurrent strictures after dilation and DVIU. Repeated endoscopic procedures can cause more complicated strictures and increase the difficulty of urethroplasty.^[2]^

The surgical reconstruction of the urethra (urethroplasty) involves various methods and techniques that may include degrees of incision or excision of the urethra with or without transferring autologous grafts or flaps.^[4]^

In the meantime, there are many tissues that can be used as grafts for anterior urethral urethroplasty surgeries such as intestinal mucosa, bladder mucosa or oral mucosa grafts especially BMG. BMG is the most commonly used graft tissue due to multiple advantages such as the easy harvesting site, being hairless, the vascular lamina propria and the richness of the elastic tissue.^[5,6]^ There is a debate over whether to close the harvesting site of the BMG or leave it to heal by secondary intention. The non-closure technique may be associated with less postoperative pain and opening difficulties. However, the existing evidence about this issue is still uncertain.^[7]^

Penile or scrotal skin can also be used as a flap. The native blood supply of the penile flap makes it more survivable and favorable for long or complicated anterior urethral strictures.^[6]^

Additionally, penile flaps are hairless and flexible and they showed decreased rates of morbidity and harvesting time compared to the grafts.^[4]^ Hair-bearing skin must not be used by surgeons for urethroplasty. The hair can obstruct the lumen and there is a risk of developing urethral calculi, recurrent urinary tract infections and a limited urine stream.^[2]^

In both choices (grafts and flaps) the success rate is high if they are performed by experienced doctors. Therefore, surgeons are free to use the tissue type and procedure that they are more familiar with.^[6,8]^

In the case of long complicated anterior structures with an unhealthy urethral palate, single-stage resection of the affected segment followed by flap or graft tabularization over a catheter has poor results.^[9,10]^ Another technique described by Morey is combining dorsal graft and ventral penile skin flap after the resection of the damaged urethral place or its augmentation by the dorsal graft.^[9]^

The combined technique provides the favorability of penile skin flaps in long strictures with the good outcomes of the dorsal BMG making it possible to complete the procedure in a single stage operation.^[9]^ The advantages of this technique are mostly due to 2 reasons: firstly, the 2 distinct blood supply sources: the BMG from the underlying tunica, which is extremely reliable, while the flap brings its own supply with it. Secondly, suturing both transferred tissues to the corpora provides more stability and possibly prevents segment contracture.^[10]^

The use of the combined graft and flap technique is being reported in the literature. Joshi et al^[11]^ described 15 cases of long bulbar and penobulbar strictures reconstructed with the graft plus penile flap technique, and with primary success rate of 86.7%. Karapanos et al^[12]^ also reported 12 patients with penile urethral strictures underwent BMG and pedicled penile skin flap transfer with an overall success rate of 91.7%.

In our case, the use of a hairy scrotal flap in the first urethroplasty and the attempt to create an extra-anatomical bypass by not overlapping the original urethral plate led to the failure of the procedure, recurrence of the symptoms and the formation of diverticulum as a complication. Therefore, surgeons who are not familiar with urethroplasty or not experienced enough should refer the patient to a more experienced urologist.^[2]^

This paper adds a new case of urethroplasty using the combined flap and graft technique, highlighting its role in the treatment of long complicated anterior urethral strictures.

## 4. Conclusion

Long anterior urethral strictures with a damaged urethral plate can be successfully reconstructed in a single-staged operation by the combination of dorsal BMG to augment the urethral plate and ventral onlay Q penile skin flap. Moreover, advice from a more expert surgeon should be taken when there is a lack of experience to avoid malpractice or complications.

## Author contributions

**Conceptualization:** Abdulrazak Anadani.

**Project administration:** Abdulrazak Anadani.

**Supervision:** M. Yasin Lutfi.

**Writing – original draft:** Abdulrazak Anadani, Aya Obaidin, Bashar Badawi.

**Writing – review & editing:** Abdulrazak Anadani.
